# Role of Small Intestinal Bacterial Overgrowth (SIBO) and Inflammation in Obese Children

**DOI:** 10.3389/fped.2020.00369

**Published:** 2020-07-07

**Authors:** Susanna Esposito, Anna Biscarini, Barbara Federici, Marta Cofini, Alberto Argentiero, Cosimo Neglia, Lucia Lanciotti, Gian Luigi de' Angelis, Nicola Principi

**Affiliations:** ^1^Pediatric Clinic, Department of Medicine and Surgery, Pietro Barilla Children's Hospital, University of Parma, Parma, Italy; ^2^Pediatric Clinic, Department of Surgical and Biomedical Sciences, Università degli Studi di Perugia, Perugia, Italy; ^3^Gastroenterology Unit, Santa Maria della Misericordia Hospital, Perugia, Italy; ^4^Unit of Gastroenterology and Digestive Endoscopy, Department of Medicine and Surgery, University of Parma, Parma, Italy; ^5^Università degli Studi di Milano, Milan, Italy

**Keywords:** chemokines, cytokines, inflammation, obesity, overweight, small intestinal bacterial overgrowth

## Abstract

Knowledge of the real incidence of small intestinal bacterial overgrowth (SIBO) in obese children and its role in obesity development seems essential for a more effective approach to the treatment of this condition. In this prospective, single-blind study, presence of SIBO was evaluated in a group of children with overweight/obesity. A blood sample for evaluation of cytokine profile was collected to establish the potential relationship with inflammatory condition and lactulose breath test (LBT) to diagnose SIBO was performed. A total of 36 patients with excess of adipose tissue were recruited. Among them, 16 (44.4%) were overweight and 20 (45.6%) were obese. Overall, 26 (72.2%) children had a positive LBT and were considered suffering from SIBO, 12 (75.0%) among those overweight and 14 (70.0%) among those obese. Measurement of cytokines (IL-1α, IL-1β, IL-4, IL-6, IL-7, IL-8, IL-10, IL-12p40, IL-12p70, IL-17, IFN-α2, IFN-γ, TNF-α), cytokine antagonists (IL-1ra), chemokines (IP10, MCP-1, MIP1α, MIP1β), and growth factors (EGF, G-CSF, GM-CSF, and VEGF) secreted in culture supernatants by PHA activated-PBMCs revealed that in the study population proinflammatory cytokines IL-1, IL-6, IL-8, IL-12, IFN-γ, IL-18, and TNF-α were high, whereas anti-inflammatory mediators IL-4 and IL-10 were low. However, no significance difference between children with SIBO and those without were evidenced. Evaluation of relationship of severity of SIBO showed a significant positive relationship between EGF or IFN-α2 and H2 but not CH4 levels and an inverse significant relationship with CH4 but not H2. Despite its limitations and further studies are needed, this study seems to indicate that SIBO is extremely common in overweight and obese children and can be demonstrated not only in severely obese subjects but also in moderately overweight patients. The inflammatory state seems to precede obesity development and SIBO does not seem to have relevance in obesity development, with no relationship found between severity of SIBO and inflammatory state.

## Introduction

Small intestinal bacterial overgrowth (SIBO) is a condition characterized by an increase in the number and/or the type of bacteria commonly detected in the upper gastrointestinal tract (UGT) (1). Generally, in UGT only small amounts of a mixture of predominantly anaerobic bacteria and some anaerobes are detected. In SIBO, bacterial density is increased and mainly anaerobes are found. Several factors can contribute to SIBO development (2). Among them, intestinal structural abnormalities (i.e., fistulae, diverticula, and primary or secondary intestinal obstructions), factors that favor bacterial selection and proliferation (i.e., achlorhydria, antibiotic use, and immunodeficiency) and, finally, factors that cause motility disorders (i.e., diabetes mellitus and scleroderma) (3).

Clinical manifestations of SIBO can vary. They can range from an asymptomatic illness or mild and non-specific intestinal symptoms (abdominal pain, diarrhea, bloating or flatulence) to a severe malabsorptive syndrome leading, particularly in children, to significant nutritional deficiencies, weight loss and growth retardation (4). However, contrarily to what could be expected, studies mainly carried out in adults have found that SIBO is more common among obese patients than in healthy non-obese individuals (5–7). This could indicate a direct relationship between SIBO and obesity. However, incidence of SIBO in obese children has not been precisely defined. Moreover, undefined are the reasons for the potential relationship between the two conditions. It could be hypothesized that, accordingly to what has been shown for lower gastrointestinal tract (LGT) dysbiosis, SIBO could impair immune system function, lead to an increased translocation of bacterial antigens toward metabolically active tissues, and cause a chronic inflammatory state. The development of obesity would be the direct consequence (8, 9). On the contrary, it could be supposed that SIBO of obese patient is only a concomitant independent condition and that nutrient overload altering metabolic homeostasis could *per se* induce a significant inflammatory state and obesity development (10).

Knowledge of the real incidence of SIBO in obese children and its role in obesity development seems essential for a more effective approach to the treatment of this condition. Presently, SIBO is treated with antibiotics (11). If it would be demonstrated that SIBO is common in obese children and it is associated with obesity development, use of probiotics, that have been found effective in reducing LGT gut dysbiosis and preventing and treating obesity (12), could be suggested in children with SIBO also. In this study, presence of SIBO was evaluated in a group of children with overweight/obesity. Moreover, to establish the potential relationship between SIBO and obesity, inflammatory condition of these patients was studied.

## Materials and Methods

### Study Design and Population

A prospective, single-blind study was carried out at the Pediatric Clinic of University of Perugia, Perugia, Italy, between August 1, 2017, and March 31, 2018. The protocol was approved by the Ethics Committee of Umbria Region (CEAS Register no. 3136/17) and the study was conducted in accordance with the standards of Good Clinical Practice for trials of medicinal products in humans. Written informed consent was obtained from the parents/legal guardian of each enrolled child and from every enrolled subject aged ≥8 years.

A total of 36 patients with excess of adipose tissue were recruited. During clinical visit age, sex, weight, height, waist circumference, and Tanner stage were recorded. History of cancer, antibiotic therapy in the last 2 weeks, chronic assumption of drugs, including proton pump inhibitors, gastrointestinal autoimmune diseases (i.e., celiac disease, inflammatory bowel diseases), were considered as main exclusion criteria. The body weight was measured using a standard balance with a variability of 0.1 kg, the height was detected with a standard wall-mounted stadiometer, with variability of 0.1 cm, the body mass index (BMI) was calculated as the ratio between weight expressed in kg and height expressed in square meters (kg/m^2^). Overweight and obese were defined as a BMI in the 85th to 94th percentiles and the 95th percentile or higher, respectively, according to the Centers for Disease Control and Prevention (13).

All subjects were evaluated for personal and family histories, dietary daily intake, physical activity and general lifestyle. Thereafter, blood examination was performed, including the complete blood count, renal and hepatic function, thyrotropin and serum free thyroxine, vitamin D serum levels, serum total cholesterol, low-density lipoprotein cholesterol, high-density lipoprotein cholesterol, and triglycerides. Blood glucose, and insulin levels and the homeostasis model assessment-estimated insulin resistance (HOMA-IR) index were checked to investigate glucose metabolism. A blood sample for evaluation of cytokine profile was also collected and lactulose breath test (LBT) to diagnose SIBO was performed.

### Determination of the Cytokine Profile

Freshly isolated PBMCs (1 × 10^6^ cells/mL) were cultured *in vitro* in RPMI medium supplemented with 10% FCS, 1 mM glutamine and penicillin/streptomycin (Gibco, Invitrogen CA, USA) at 37°C in a humidified 7% CO2 incubator, before harvesting culture supernatants for analysis. For assessment of cytokine production, T1D PBMCs were activated *in vitro* with 1 μg/mL phytohemagglutinin (PHA; Sigma-Aldrich, MO, USA) for 48 h, before harvesting culture supernatants. A 15-plex immunoassay (Bio-Plex Pro Human Th17 cytokine panel, Cat N. 171aa001m; Bio-Rad, CA, USA) and a MAGPIX system (Luminex Corporation) were used for the detection of cytokines. Cytokine levels were represented as the mean ± standard deviation (SD) of the concentrations (pg/mL) and analyzed by two-tailed unpaired Student's *t*-test (probiotic vs. control).

### Lactulose Breath Test (LBT)

The preparation scheme included: no antibiotics, lactic ferments, laxatives to be taken 10 days prior to the test; a diet followed the day before the test taking only tea for breakfast, boiled rice seasoned with oil, meat or grilled fish or boiled at lunch and dinner.

The test was then performed by taking fine exhalation air samples under basal conditions and after the lactulose, in water solution, administration. The breath test started by collecting a baseline sample of H2 and CH4 levels of the patient's breath to be examined. Thereafter, the patients drunk a solution of lactulose based on the physician's choice. The patient breaths into a breath analyzer (QuinTron, QuinTron Instrument Company, USA) every 15 min for the following 3 h and finally the breath samples were examined.

LBT was defined positive with an increasing evidence of either H2 or CH4 at least 20 ppm (parts per million) within the first 90 min.

### Statistical Analysis

All statistical analyses were performed by using Stata Statistical Software: Release 12 (StataCorp LP, College Station, TX, USA). Quantitative variables were reported as mean ± SD and dichotomous variables such as presence or absence of a condition were reported as frequency and percentage. Mean differences for quantitative variables between males and females and LBT positive and LBT negative groups were assessed by Wilcoxon rank-sum test. Differences between groups for dichotomous variables were evaluated by Fisher's exact test when the frequency values were less than or equal to 5 and by the chi-square in the other cases. Pearson's correlation coefficients were calculated to evaluate the association between tested mediators and H_2_ and CH_4_ measurements.

## Results

Table 1 shows general characteristics of the 36 enrolled children. Among them, 16 (44.4%) were overweight and 20 (45.6%) were obese. A total of 26 (72.2%) children had a positive LBT and were considered suffering from SIBO, 12 (75.0%) among those overweight and 14 (70.0%) among those obese. Comparison of children with and without SIBO revealed that no difference between groups was demonstrated in age (11.4 ± 2.00 yrs in those with SIBO vs. 10.6 ± 2.20 yrs in those without) and gender distribution (53.8% of males in those with SIBO and 40.0% in those without) as well as BMI (28.1 ± 4.7 kg/m^2^ vs. 26.2 ± 2.90 kg/m^2^). Personal and family histories as well as laboratory values were similar between patients with SIBO and those without.

**Table 1:** General characteristics of study population stratified by gender.

	**All *N =* 36**	**Females *N =* 18**	**Males *N =* 18**
Age, years	11.1 ± 2.1	11.3 ± 2.1	11.1 ± 2.1
BMI, mean ± SD	27.7 ± 4.4	27.5 ± 4.3	28.0 ± 4.5
BMI percentiles	95.8 ± 4.2	95.3 ± 4.8	96.2 ± 3.7
LBT negative, n (%)	10 (7.8)	6 (33.3)	4 (22.2)
LBT positive, n (%)	26 (72.2)	12 (66.7)	14 (77.8)

*BMI, body mass index; LBT, lactulose breath test. No significant difference between the groups*.

Table 2 summarizes levels of cytokines, cytokine antagonists, chemokines, and growth factors secreted in culture supernatants by PHA activated-PBMCs according to SIBO status. Measurement of levels of cytokines (IL-1α, IL-1β, IL-4, IL-6, IL-7, IL-8, IL-10, IL-12p40, IL-12p70, IL-17, IFN-α2, IFN-γ, TNF-α), cytokine antagonists (IL-1ra), chemokines (IP10, MCP-1, MIP1α, MIP1β), and growth factors (EGF, G-CSF, GM-CSF, and VEGF) revealed that in the study population proinflammatory cytokines IL-1, IL-6, IL-8, IL-12, IFN-γ, IL-18, and TNFα were high, whereas anti-inflammatory mediators IL-4 and IL-10 were low. However, no significance difference between children with SIBO and those without were evidenced.

**Table 2:** Levels of cytokines, cytokine antagonists, chemokines, and growth factors secreted in culture supernatants by PHA activated-PBMCs according to small intestinal bacterial overgrowth (SIBO) status.

	**Without SIBO *N =* 10**	**With SIBO *N =* 26**
EGF, pg/mL	34.1 ± 7.3	55.5 ± 36.3
G-CSF, pg/mL	22.5 ± 8.4	36.0 ± 20.0
GM-CSF, pg/mL	4.2 ± 1.2	13.7 ± 13.4
IFNα2, pg/mL	23.8 ± 4.3	38.1 ± 22.1
IFNγ, pg/mL	17.8 ± 7.9	16.3 ± 4.6
IL-10, pg/mL	17.9 ± 9.7	20.1 ± 9.4
IL-12p40, pg/mL	66.0 ± 31.8	66.6 ± 12.8
IL-12p70, pg/mL	8.6 ± 3.0	8.4 ± 2.7
IL-17, pg/mL	9.3 ± 0.8	6.4 ± 2.7
IL-1ra, pg/mL	52.1 ± 25.7	66.3 ± 19.7
Il-1α, pg/mL	8.4 ± 0.6	9.6 ± 2.8
IL-1β, pg/mL	16.7 ± 3.8	11.1 ± 6.0
IL-4, pg/mL	13.5 ± 5.4	13.0 ± 5.3
IL-6, ng/mL	33.4 ± 36.8	32.4 ± 26.6
IL- 7, ng/mL	7.2 ± 1.0	8.6 ± 3.7
IL-8, ng/mL	2,455.3 ± 1,805.0	2,285.0 ± 1,918.2
IP10, ng/mL	48.7 ± 16.4	62.9 ± 49.3
MCP-1, ng/mL	4,298.0 ± 2,651.8	4,303.8 ± 3,656.6
MIP-1α, ng/mL	63.2 ± 36.5	52.7 ± 29.9
MIP-1β, ng/mL	233.7 ± 203.6	201.4 ± 153.3
TNF-α, ng/mL	25.2 ± 16.2	21.2 ± 16.1
VEGF, ng/mL	64.8 ± 15.5	100.1 ± 55.5

*No significant differences between the groups*.

Evaluation of relationship of severity of SIBO measured by concentration of expired H2 and CH4 during LBT with concentrations of all tested soluble mediators showed a significant positive relationship only between EGF or IFN-α2 and H_2_ but not CH_4_ levels and an inverse significant relationship with CH_4_ but not H_2_ (Table 3). No significant association could be observed for all the other studied mediators.

**Table 3:** Pearson significant correlations of H_2_ and CH_4_ values and cytokines secreted in culture supernatants by PHA activated-PBMCs.

	**H_**2**_**	**CH_**4**_**
EGF	*r* = 0.52 *p* < 0.05	*r* = −0.01 *p* = 0.99
IFNα2	*r* = 0.50 *p* < 0.05	*r* = −0.01 *p* = 0.96
MIP-1α	*r* = −0.30 *p* = 0.25	*r* = 0.50 *p* < 0.05

## Discussion

This study seems to indicate that SIBO is extremely common in overweight and obese children. More than two thirds of the pediatric patients enrolled in this research had increased hydrogen and/or methane breath concentrations after LBT. Moreover, this study highlights that SIBO can be demonstrated not only in severely obese subjects but also in moderately overweight patients.

The prevalence of SIBO evidenced in this research is quite similar to that recently found by Roland et al. (7) in a group of obese adults and even higher than that reported in previous studies (5, 6, 14). Initially, it was reported that an association with SIBO could be evidenced when BMI was > 40 kg/m^2^ (5). Later, it was shown that SIBO could be demonstrated in patient with BMI lower than 30 kg/m^2^ (7). In our study population, a few children were simply overweight with BMI only slightly higher than 25 kg/m^2^. However, none of them showed non-alcoholic fatty liver disease (NAFLD)/ non-alcoholic steatohepatitis (NASH).

In all the children enrolled in this study, independently of the presence of SIBO, a significant inflammatory condition was evidenced. Levels of all the markers of inflammation that have been tested in this study were above the normal limits in both children with and without SIBO. This seems to indicate that factors other than SIBO can favor obesity and SIBO seems an accidental condition that can occur in overweight/obese children. Moreover, the demonstration of a significant inflammatory condition even in children with moderate overweight suggests that the inflammatory state precedes obesity development and SIBO does not seem to have relevance in obesity development. The evidence that no relationship was found between severity of SIBO and inflammatory state seems to further support this conclusion. However, before establishing that SIBO has no role in favoring obesity development, it must be excluded that microbial diversity in the LIT can be one of the most important reasons for obesity development. At this regard, a previous study showed that rectal samples collected in obese and not obese subjects with SIBO revealed significant differences in microbiota composition, mainly for decreased microbial diversity in obese patients (12).

This study has several limitations. First, the sample of study patients is small. Secondly, despite hydrogen breath tests are currently used for the diagnosis of SIBO in clinical practice based on their non-invasive nature, low cost, and technical and logistical simplicity, their accuracy is debated. Compared to upper gut aspirate (i.e., the gold standard for SIBO diagnosis), both glucose breath test (GBT) and LBT have limitations. GBT is highly specific (78–97%) but has been found poorly sensitive (15.7–62%). False negative results are not uncommon, as glucose can be completely absorbed in the proximal small bowel and may not reach the site of SIBO (15, 16). To reduce this risk, LBT was used in this study, although this test is not without criticism. Conventional double-peak criteria on LHBT lack sensitivity (31–68%) and the recently proposed early-peak criterion, the one used in this study, can lead to false positive results with specificity of 65–97.9% (17, 18). Thirdly, the study did not include a control group of non-overweight/obese children with SIBO in whom inflammatory markers have been studied. All these limitations highlight that the results of this study should be confirmed by further studies able to definitively establish whether SIBO is only an occasional finding in overweight/obese children or play a real role in causing BMI increase The use of molecular microbiological methods to the characterization of intestinal microbiome in both obese and normal weight children could be helpful to clarify these results (19).

## Conclusions

This study highlights that SIBO detected with LBT is extremely common in overweight and obese children and can be demonstrated not only in severely obese subjects but also in moderately overweight patients. The inflammatory state seems to precede obesity development and SIBO does not seem to have relevance in obesity development, with no relationship found between severity of SIBO and inflammatory state. Considering the small number of children enrolled, the limitations of the method used to diagnose SIBO and the lack of a control group, conclusions of this study must be evaluated carefully. Further studies are needed to verify whether the use of probiotics may be useful in reducing inflammation in addition to gut dysbiosis in children and adolescents with overweight and obesity.

## Data Availability Statement

All datasets generated for this study are included in the article/supplementary material.

## Ethics Statement

The studies involving human participants were reviewed and approved by Ethics Committee of Umbria Region. Written informed consent to participate in this study was provided by the participants' legal guardian/next of kin.

## Author Contributions

SE wrote and supervised the project and wrote the first draft of the manuscript. AB, MC, and LL were in charge of the patient's follow-up. BF performed lactulose breath test. AA and CN were in charge of data management and statistical analyses. GA validated laboratory analyses. NP provided scientific contributions and critically revised the paper. All authors contributed to the article and approved the submitted version.

## Conflict of Interest

The authors declare that the research was conducted in the absence of any commercial or financial relationships that could be construed as a potential conflict of interest.
